# Social Action Effects: Representing Predicted Partner Responses in Social Interactions

**DOI:** 10.3389/fnhum.2022.837495

**Published:** 2022-06-02

**Authors:** Bence Neszmélyi, Lisa Weller, Wilfried Kunde, Roland Pfister

**Affiliations:** Department of Psychology, University of Würzburg, Würzburg, Germany

**Keywords:** motor control, action effects, action representation, sociomotor control, ideomotor theory

## Abstract

The sociomotor framework outlines a possible role of social action effects on human action control, suggesting that anticipated partner reactions are a major cue to represent, select, and initiate own body movements. Here, we review studies that elucidate the actual content of social action representations and that explore factors that can distinguish action control processes involving social and inanimate action effects. Specifically, we address two hypotheses on how the social context can influence effect-based action control: first, by providing unique social features such as body-related, anatomical codes, and second, by orienting attention towards any relevant feature dimensions of the action effects. The reviewed empirical work presents a surprisingly mixed picture: while there is indirect evidence for both accounts, previous studies that directly addressed the anatomical account showed no signs of the involvement of genuinely social features in sociomotor action control. Furthermore, several studies show evidence against the differentiation of social and non-social action effect processing, portraying sociomotor action representations as remarkably non-social. A focus on enhancing the social experience in future studies should, therefore, complement the current database to establish whether such settings give rise to the hypothesized influence of social context.

## The Sociomotor Approach to Social Interactions

Human interaction is action. It necessarily involves the generation of efferent activity of the involved partners, like gesturing, facial expressions, eye movements, or speaking. The question of how the generation of such efferent activity depends on a social interaction context is a key question in contemporary research on human motor control (Wolpert et al., 2003; Knoblich et al., 2011). Here we adopt the perspective of sociomotor action control, a theoretical framework that focuses on the impact of action-contingent events in the agent’s social environment (Kunde et al., 2018). The sociomotor framework rests on classical ideomotor theorizing by assuming that actions are represented and controlled in terms of their perceivable effects (Harleß, 1861; James, 1890). When interacting with the inanimate environment, these effects include body-related signals such as proprioceptive changes produced by the moving limb (Rowe, 1910; Pfister, 2019) and movement-contingent events in the agent’s body-external environment (Kunde, 2001; Hommel, 2009). In ideomotor theorizing, action-effect anticipation, i.e., the mental recollection of previous action effects, is a means to select and initiate body movements, a process that is made possible by bi-directional associations between sensory effects and motor activity that had preceded the effect.

**Figure 1:**
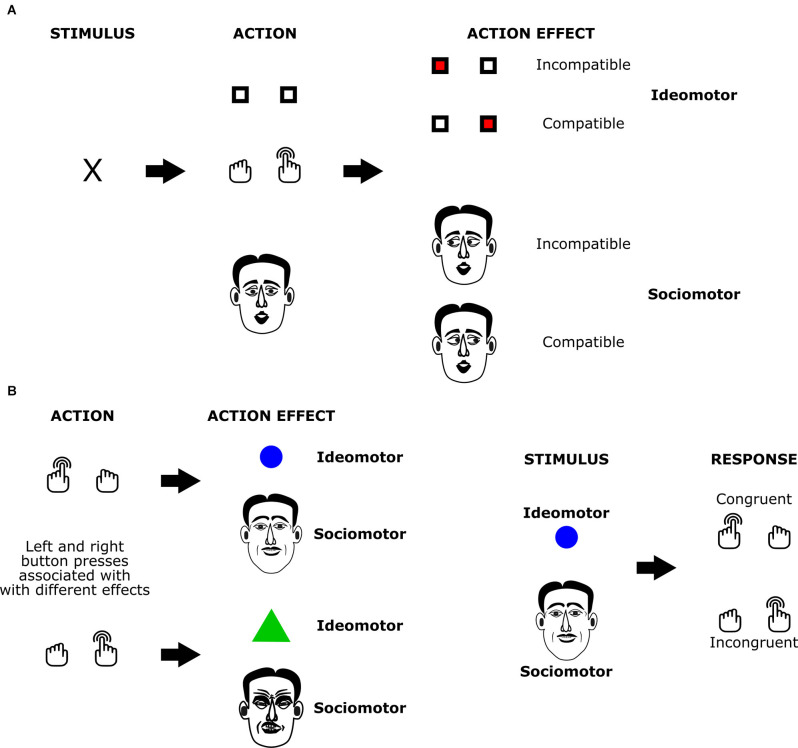
The structure of ideomotor paradigms with social and non-social effects. In the *response effect compatibility paradigm* **(A)** actions elicit effects with either matching (compatible condition; here: right button presses trigger stimuli presented on the right side of the screen or rightward eye movement of the partner) or non-matching features (incompatible condition; here: right button presses trigger stimuli presented on the left side of the screen or leftward eye movement of the partner). Studies compare action initiation times for actions with compatible and non-compatible effects. In the acquisition phase of the *two-stage learning* paradigm **(B)**, participants learn associations between actions and elicited effects (here: left and right button presses are associated with different geometric shapes or with different facial expressions). In the subsequent test phase, participants have to execute responses after stimuli that served as action effects in the acquisition phase are presented to them. Studies compare response times or choice frequencies for stimulus-response pairings that are congruent and incongruent with the previously acquired action-effect associations.

The sociomotor framework is based on the assumption that action-triggered changes in the social environment can also play a role in ideomotor action control processes. The most common variant of such social action effects are movements that co-actors perform in response to an agent’s own action: when we greet a friend arriving at the airport by waving at them, they usually respond by performing the same action. At other times, we do not perceive the movements of the person directly but only the remote effects of these reactions, e.g., when ringing the doorbell and hearing the lock being opened. Whereas common ideomotor accounts do not assume a special role of social action effects, the sociomotor framework departs from this “conservative” assumption by highlighting likely peculiarities of social interactions as compared to interactions with the inanimate environment (e.g., delayed and inconsistent effects, the similarity of action, and effect). Early studies in this area have focused on demonstrating that bi-directional associations between actions and their social effects (i.e., the behavior of others in response to these actions) are established as assumed by the sociomotor framework (e.g., Herwig and Horstmann, 2011; Pfister et al., 2013; Müller, 2016). These studies usually adapted tried-and-tested paradigms of ideomotor research to social settings: studies on *response-effect compatibility* (Kunde, 2001; Kunde et al., 2004; Pfister and Kunde, 2013) suggest that whenever actions and intended, action-contingent events vary on a shared dimension, action planning, and initiation is efficient in case of matching features on this dimension as compared to non-matching features. In *two-stage learning* studies (Hommel, 1996; Elsner and Hommel, 2001; Pfister et al., 2011), participants first establish action-effect associations in an acquisition phase. In a subsequent test phase, the effects of the first session are presented before the response to measure effect-induced response tendencies *via* reaction times or choice frequencies. Sociomotor studies have leveraged these designs by replacing the inanimate effects of the initial methods with social effects (Figure 1). Response-effect compatibility phenomena and learning effects generalized to these social settings, showing that action representations indeed incorporate action effects in the agent’s social environment.

Sociomotor studies usually utilize a leader-follower setup with one participant initiating the interactions, and a second participant reacting to these actions by performing predetermined responses. Tasks involve direct face-to-face interactions between two participants or interactions with a virtual co-agent (e.g., Herwig and Horstmann, 2011; Pfister et al., 2013, 2017; Lelonkiewicz et al., 2020). In the latter case, the follower’s response is replaced with stimuli representing the reactions of a human partner, e.g., images of facial expressions or animated body movements. As a starting point, higher level goals and mutual coordination of movements that play a role in both everyday social interactions and in joint action research (Vesper et al., 2010; Sacheli et al., 2015) were typically excluded by design, and are yet to be implemented in this framework (for a recent step in this direction, see Müller and Jung, 2018; Müller, 2020). Since the action options of both participants are limited to a large degree by the experimental setup and corresponding instructions, this approach usually results in situations that do not capture all facets of social interaction proper. However, in more naturalistic settings, the influence of the link between action and its social effect is difficult to separate from other factors that contribute to the planning and execution of actions. In those interactions, several parallel but interconnected predictive models might be involved (Pesquita et al., 2018), and it is difficult to isolate their contributions to individual action control. The reductionist approach of the sociomotor framework, on the other hand, focuses on the link between the action of the leader and the reaction of the follower, which makes it possible to assess the influence that predictions regarding the partners’ responses to one’s own actions have on the planning and execution of actions.

While early sociomotor studies have provided proof-of-principle evidence for the role of social action effects on human action control, lately, attention shifted towards factors that may distinguish control over social as compared to non-social events. As we will demonstrate in the following sections, currently, differences between interactions with the social and with the inanimate environment are less apparent in sociomotor paradigms than in other areas that are related to action-effect binding (e.g., sense of agency and temporal binding: Stephenson et al., 2018; Silver et al., 2021; Vogel et al., 2021). Thus, the goal of the current review is to re-orient research on sociomotor action control by incorporating insights from related fields of study.

## Challenges of Social Action-Effect Representation

Predictability of action effects arguably is a key component of any action control mechanism (e.g., Watson, 1997). Efficient action selection requires that the effects associated with various action options can be accurately predicted, and the same holds true for action planning and initiation *via* ideomotor mechanisms. Social contexts seem to pose a special challenge in this regard. First, social interactions are usually more “noisy” than interactions with the inanimate environment: large and variable action-effect delays and inconsistent action effects are commonplace in social settings since the identity and timing of social action effects depend on reactions by a human interaction partner (Kunde et al., 2018). Second, social effects are often complex stimuli. Current ideomotor theorizing suggests that actions are represented in a distributed manner (Hommel et al., 2001), meaning, it is not the action effects as a whole that must be predictable, but features that can effectively distinguish between various action options. However, complex stimuli can be described along multiple feature dimensions, so identifying relevant dimensions of social effects can be a difficult task. And yet, we seem to complete such tasks effortlessly. For example, a study by Herwig and Horstmann (2011)—showing that saccades were directed more towards the mouth region when an action (eye movement) elicited a smiling face and more towards the eyebrow region when a frowning face was generated by the action—indicated that even in the case of complex facial responses, participants were able to select and predict relevant features that conceivably mediate their own action production. In the following we suggest two hypotheses that could separate action control processes involving social effects from common ideomotor mechanisms and that might explain how sociomotor action representations accommodate the challenges described above: (1) *via* the representation of additional feature dimensions and (2) *via* attentional pre-selection.

Studies that use social stimuli as action effects convincingly show that despite the challenges detailed in the previous section, social effects are integrated into action representations and can contribute to the planning and initiation of movements. However, it is an open question whether—or under what conditions—the role of social action consequences differs from that of inanimate effects. Studies that addressed this question directly do not provide a clear picture. Some did not observe any differences in the utilization of social and non-social effects: For instance, Neszmélyi and Horváth (2021) reported a similar influence of auditory action effects on the force of the movements when tones were elicited directly and when they were produced in a leader-follower setup with the follower being directly responsible for generating the tones. A similar pattern was also observed with action initiation times in a study investigating spatial compatibility effects: compatible action effects boosted performance compared to incompatible effects to the same degree, irrespective of whether these effects were produced with or without reliance on the responses of a second agent (Experiment 1 and 2 in Müller, 2016). Although these studies confirm that social action effects contribute to action control, they do not support the idea that specific social features of the stimuli would be represented, but rather suggest that social effects are also represented in a non-social manner. However, other studies reported effects that seem unique to social stimuli, indicating differences in the contribution of social and inanimate effects to action control: For example, Kunde et al. (2011) observed a faster generation of facial gestures (smiling, frowning) that foreseeably triggered the presentation of faces with corresponding rather than non-corresponding gestures, while this was not observed when own facial gestures triggered inverted faces. Flach et al. (2010) reported a directional compatibility effect that was only observed with social effects, that is, when the initiation of a handshake resulted in the image of a hand pointing towards the participant’s hand (representing the acceptance of the handshake). For non-social action consequences (i.e., when the handshake-initiating actions elicited arrow stimuli), the influence of directional compatibility was not significant.

In the studies of Kunde et al. (2011) and Flach et al. (2010), social stimuli offer a particular type of feature that is not encountered in interactions with the inanimate environment: Anatomical information that maps directly onto the bodily makeup of the agent (see also Colton et al., 2018). Although these studies do not demonstrate the representation of anatomical features directly, they show that in some cases, when non-social effects do not have an influence on action selection, action effects that can be characterized by anatomical features are still involved in motor control processes. The representation of these features could plausibly solve issues related to the complexity of social effects. When relevant differences between action effects are difficult to capture with basic spatiotemporal dimensions, anatomical coding might provide a simpler solution. The visual representation of bodily movement is necessarily specific to either own actions or actions of social interaction partners, which can be expected to exert a unique impact on action control (Kunde et al., 2018). This is especially plausible in light of current theories on action observation, which usually assume that using one’s own motor system to simulate observed movements is a key component in interpreting other people’s actions and making predictions about the outcome of these actions (Knoblich and Flach, 2001; Keller et al., 2007; Sacheli et al., 2021). Using similar simulations for partners’ reactions could help predict the effectors that they will use to execute their responses. Thus, it could support the extraction of anatomical features during the acquisition of action-effect associations and it could also enhance effect anticipation, thereby contributing to the selection and initiation of actions. A mechanism based on action simulation would also be well suited to handle delays inherent in social interactions: simulation could help in estimating the time that it takes to perform a response and form relatively accurate predictions about action-response delays.

Indirect support for anatomical coding in sociomotor action control comes from studies on automatic imitation (Catmur and Heyes, 2011; Boyer et al., 2012). Here, observing a finger movement was shown to prime actions that shared either spatial or anatomical features, with independent contributions of both feature types. Interestingly, not only actually perceived actions of the model but also their predicted actions can induce the execution of corresponding movements in the observer: while observing videos about people who had their hair falling into their face, or who were wrinkling their nose, participants showed an increased tendency to perform actions related to the relevant body part (hair stroking, nose scratching) even though these actions were never performed by the model (Genschow and Brass, 2015; Genschow et al., 2018). This shows that not only observed movements can activate actions but also stimuli that share their anatomical features. In imitation studies, the impact of social events was mediated by stimulus-response and not by action-effect associations, thus, it is an open question to what extent such effects could also contribute to sociomotor action control as captured in corresponding paradigms. Since imitation and action initiation by effect anticipation might be closely related (Bunlon et al., 2015), these observations still suggest that a similar role for anatomical features might also apply to action-effect binding.

A recent direct test of the hypothesized role of anatomical features returned surprisingly negative results, however (Weller et al., 2019). It has been observed previously that actions that are imitated by a partner in a leader-follower setup, are initiated faster than counterimitated movements (Pfister et al., 2013; Müller, 2016; Lelonkiewicz et al., 2020). In the study of Weller and colleagues, followers, seated opposite the leaders, were imitating the leaders’ right-handed finger movements with either their right hand (spatial and anatomical compatibility are separated and have opposite effects) or their left hand (spatial and anatomical compatibility overlap). The results indicated that spatial and anatomical compatibility jointly affected the follower’s performance (confirming previous findings on action imitation), but only spatial compatibility had a significant influence on action initiation for the leader. The latter finding portrays sociomotor action representations as surprisingly non-social in the sense that they mainly draw on features that also characterize events in the non-social environment.

At the same time, the absence of anatomical feature representation in this study does not necessarily generalize across different types of interactions. First, anatomical features might only be represented under certain conditions (Weller et al., 2019). There is arguably a difference in the contribution of various effect features to action control (Mocke et al., 2020). The weighting of features could be influenced by factors like task relevancy or saliency. It is possible that the contribution of anatomical features to effect-based control processes would be apparent in tasks where, due to coordination demands or interaction costs, the representation of anatomical features is salient or relevant. Second, the setting of the study (participants seated facing each other, with their hands aligned) might have primed spatial coding. In real interactions, the spatial relation of participants is usually dynamically changing, and in such cases, anatomical representations arguably could be more useful than spatial coding. Thus, the role of anatomical features might be stronger in settings where spatial feature representation is not dominant (e.g., seating participants orthogonally instead of opposite each other: Bunlon et al., 2015). Approaching these issues will certainly allow for a more definite answer on the role of anatomical features for sociomotor action representations.

Even if future studies were to confirm an absent contribution of anatomical features to effect-based action control, prioritization of socially relevant information could still distinguish social effects from inanimate action consequences: challenges caused by the perceptual complexity of social effects and by the presence of irrelevant effect dimensions could be solved by an attentional pre-selection mechanism (Herbort and Butz, 2012). The goal of this mechanism would be to orient attention towards relevant feature dimensions or towards parts of the stimulus where features that can distinguish between various effects are most likely to appear. If attentional processes limit the number of feature dimensions that are available for action-effect binding and increase the relative salience of relevant dimensions during social interactions, basic stimulus features might be sufficient for representing actions, making the representation of additional features redundant. Identifying the relevant features of social effects might also be aided by the action simulation mechanism discussed in the previous section. Attentional pre-selection that supports quick identification of relevant features could aid in faster integration of such features into action representations (Herbort and Butz, 2012) and it could also increase the weight of those features in the action representations (Hommel, 2004).

Tentative support for the pre-selection account comes from studies on monitoring the social as compared to non-social action effects (Pfister et al., 2020). Here, participants either interacted with a social partner or with a computer and were confronted with either partner errors or machine malfunctions from time to time. These oddball events systematically slowed down the immediately following response of the observing participant, replicating previous demonstrations of post-oddball slowing (Notebaert et al., 2009; Saunders and Jentzsch, 2012). The slowing was more pronounced in the case of social effects as compared to effects in the inanimate environment, indicating that more attentional resources are allocated to the processing of social effects, which is consistent with the attentional pre-selection hypothesis.

## Future Directions

Disentangling the anatomical feature representation and attentional pre-selection hypotheses is challenging. However, there are a few points where predictions based on the two ideas diverge. For example, if anatomical features can be part of the action representations, binding them with other features into an event representation should make them more difficult to access for perceptual processing (Schütz-Bosbach and Prinz, 2007). Thus, an experiment complementing social action effect production by a secondary task that requires the perceptual processing of stimuli that share features with the action effects could be a future option to contrast the two approaches.

More importantly, however, identifying circumstances where differences can be observed in the utilization of social and non-social effects could also contribute to a better understanding of the role of social factors in effect-based control processes. The lack of social influence in previous sociomotor studies might be explained by their conservative methodological approach of using reductionist paradigms to isolate effect-related action control processes from other factors that could have an impact on action parameters during social interactions (e.g., communication of intention between participants; Pezzulo et al., 2013; Sacheli et al., 2013; Vesper et al., 2017; Grynszpan et al., 2019). Yet, factors like shared goals (Vesper et al., 2010; Sacheli et al., 2013), the possibility of communicating intentions, intention sharing (Knoblich et al., 2011), or reciprocity might be necessary prerequisites of a genuine social experience and limiting their influence might contribute to the lack of unique social effects in some of the sociomotor studies. An important goal of future research therefore should be to assess the influence of social effects with new methods that incorporate factors that could enhance the experience of social interaction (Table 1).

**Table 1:** Factors that influence the social experience in interactive tasks.

Task sharing vs. shared goals	Studies often highlight goal sharing as an essential building block of joint action (e.g., Vesper et al., 2010; Sacheli et al., 2021). Working towards a shared goal might separate real interactions from actions that are perceived as independent despite causal links between them. In previous sociomotor paradigms, however, such higher-level goals are often neglected: leader’s actions are interpreted as being solely aimed at eliciting a response from the follower, while the followers are to execute predetermined responses without any consideration, reducing their role to that of a “human light switch” (Müller, 2016).
Intention communication	When verbal channels are limited, co-actors might communicate their intentions through actions that they perform in the shared task. For example, when carrying furniture up the stairs, grabbing an item at the top or at the bottom can signal the intention to lead or to follow when going up the stairs. The possibility of communication through actions might be required to perceive the context as social and process the action effects accordingly (Grynszpan et al., 2019).
Well-defined vs. dynamic leader and follower roles	In most real-life situations, leader and follower roles are not as clearly defined as in sociomotor tasks. During interactions, a change of roles might occur. Furthermore, roles might not be predetermined, but evolve dynamically as the result of intention communication (see previous point). Limiting such natural dynamics might negatively affect the social experience.
Action-effect delays	Generally, action-effect delays are longer in social interactions than in interactions with the inanimate environment. This is, however, not always considered in studies on social action effects.
Action-effect contingency: errors and free choice	Human partners are expected to commit errors. If there is a perfect contingency between leaders’ and followers’ actions, the experience of social interaction might not be elicited. It is possible that in such cases, leaders do not distinguish between social and non-social action effects.
Output modalities	Some effector systems are used primarily to affect other people’s behavior, while others are used both when interacting with other humans and with the inanimate environment (Kunde et al., 2018). Using a dedicated “social” effector for generating action effects might affect the impact of the social context on action-effect binding, although to our knowledge there is currently no published work on this topic yet.
Input modalities	Specialized systems are activated when observing human movement (Thompson, 2005; Kilner et al., 2007; Elsner et al., 2012). These systems might be also involved in action effect prediction when human movements are the consequences of one’s own actions. However, not all social effects are perceived as human movement: in some cases, only the effects produced by the co-actor are available and the movement itself cannot be perceived. Although systems dedicated to the processing of human movement might also be active when only perceiving the effects of a movement, there could be differences in action-effect binding when social effects are perceived as human movement and when they appear in a similar way as inanimate effects.

Taken together, ideomotor-inspired research in social settings has been successful in showing that social effects are utilized during movement planning and initiation. What the current database does not allow is the crucial question of whether there is a unique social aspect to sociomotor action control. On the one hand, indirect evidence suggests that social action effect representation might harness specific resources, like action simulation, and this could affect both the content of action representations and the efficiency of motor control mechanisms that rely on action effect anticipation. On the other hand, currently available data do not provide strong grounds to argue for the hypothesized influence of social context, and in several sociomotor studies, similarities in the role of social and non-social action effects are more apparent than differences between them. Results that indicate a non-social representation of social action consequences seem surprising against the background of classic findings from joint action and automatic imitation, which indicate a strong impact of social factors on action control (Kilner et al., 2003; Sebanz et al., 2006; Tsai et al., 2008; Liepelt and Brass, 2010; Sahaï et al., 2019; Sebanz and Knoblich, 2021). Deciding whether this apparent contradiction resembles a genuine difference between the processes investigated in these fields or whether it is fueled by the specific approach of previous sociomotor paradigms requires a paradigm shift in the latter field. Drawing on methods that are utilized in the field of joint action research, the sociomotor approach could provide further valuable contributions by either yielding evidence for unique social aspects or by gathering additional evidence why this area of social action control might indeed be remarkably non-social in nature.

## Author Contributions

BN, LW, WK, and RP contributed to conception of the core ideas. BN wrote the first draft of the manuscript. All authors contributed to the article and approved the submitted version.

## Conflict of Interest

The authors declare that the research was conducted in the absence of any commercial or financial relationships that could be construed as a potential conflict of interest.

## Publisher’s Note

All claims expressed in this article are solely those of the authors and do not necessarily represent those of their affiliated organizations, or those of the publisher, the editors and the reviewers. Any product that may be evaluated in this article, or claim that may be made by its manufacturer, is not guaranteed or endorsed by the publisher.
